# *Mycoplasma pneumoniae* Monoclonal P1 Type 2c Outbreak, Russia, 2013

**DOI:** 10.3201/eid2202.151349

**Published:** 2016-02

**Authors:** Inna Edelstein, Svetlana Rachina, Arabella Touati, Roman Kozlov, Nadège Henin, Cécile Bébéar, Sabine Pereyre

**Affiliations:** Smolensk State Medical University of Ministry of Health of Russian Federation, Smolensk, Russian Federation (I. Edelstein, R. Kozlov);; Inter-regional Association for Clinical Microbiology & Antimicrobial Chemotherapy, Smolensk (S. Rachina);; University of Bordeaux, Bordeaux, France (A. Touati, N. Henin, C. Bébéar, S. Pereyre);; Institut National de la Recherche Agronomique, Bordeaux (A. Touati, N. Henin, C. Bébéar, S. Pereyre);; Bordeaux University Hospital, Bordeaux (C. Bébéar, S. Pereyre)

**Keywords:** Mycoplasma pneumoniae, bacterial typing, infectious disease outbreaks, Russia, variant 2c, bacteria, respiratory infections, pneumonia\

**To the Editor:** *Mycoplasma pneumoniae* is a major cause of respiratory infections among children and young adults and is responsible for up to 40% of all community-acquired pneumonia. In 2011, an epidemic of *M. pneumoniae* infection was reported in several countries in Europe and Asia and in Israel that primarily involved adhesin P1 type 1 strains and only a few P1 type 2 strains (*1*,*2*). The spread of *M. pneumoniae* was polyclonal (*1*–*3*), except in a few semiclosed settings, such as schools (*4*). Recently, a new adhesin P1 type 2 variant, named 2c, was reported (*5*,*6*) and accounted for 8.3% of 96 *M. pneumoniae*–positive samples in Germany (*7*).

In 2013, an increase in the number of community-acquired pneumonia cases was reported in children and their adult contacts from 2 towns in Russia separated by 45 km, Ozerniy and Duchovshina, during January–March and October–November, respectively. To characterize the outbreak in Ozerniy, we collected 13 throat swabs from 9 symptomatic children and 4 asymptomatic adults who were the parents or grandparents of the affected children. All children attended the same school and were treated in the same district hospital as inpatients or outpatients. In Duchovshina, throat swab samples were collected from 17 children and 2 adults. The children attended the same school, and the preschool-aged children visited the same daycare center 1 km away. One adult patient was the first aid driver who transported the children to the hospital. The other adult patient was a community center worker who spent time with the children. In both cities, the symptomatic patients received β-lactams as initial therapy before testing.

All specimens were processed in the laboratory of molecular diagnostics of the Smolensk State Medical Academy (Smolensk Russia). Nucleic acids were extracted by using the DNA-sorb-AM nucleic-acid extraction kit (InterLabService, Moscow, Russia), and *M. pneumoniae* was subsequently detected by using the AmpliSens *Mycoplasma pneumoniae/Chlamydophila pneumoniae*-FRT PCR kit (InterLabService). Two *M. pneumoniae* molecular typing methods, adhesin P1 typing and multilocus variable-number tandem-repeat analysis (MLVA), were performed as previously described (*1*,*5*,*7*). Macrolide resistance-associated mutations were searched using real-time PCR and melting curve analysis (*1*).

The *M. pneumoniae* isolates from the specimens collected in Ozerniy were all adhesin P1 type 2c and belonged to 4 distinct MLVA types, 1 of which, MLVA type 73563, has not been previously reported (Table). Without including the instable MPN1 marker (*8*), we observed only 2 MLVA types. The 19 *M. pneumoniae* isolates from the specimens collected in Duchovshina also were all P1 type 2c, and all belonged to the same MLVA type, 43562 (type M). No macrolide resistance–associated mutation was observed in any city. 

**Table:** Characteristics of 61 *Mycobacterium pneumoniae*–positive respiratory tract specimens collected in Ozerniy, Duchovshina, and the Smolensk region, Russia*

City or region and specimen designation	Sample source	Patient age, y	Hospitalization status	Respiratory clinical syndrome	Date of collection	MLVA type†	MLVA type without MPN1‡	PCR-RFLP type	Macrolide resistance genotype
Ozerniy									
38795	Throat swab	12	Inpatient	Pneumonia	2013 Feb 15	53562 (S)	3562	2c	Wild type
38796	Throat swab	10	Inpatient	Pneumonia	2013 Feb 15	73562 (Y)	3562	2c	Wild type
38799	Throat swab	10	Inpatient	Pneumonia	2013 Feb 15	73563	3563	2c	Wild type
38812	Throat swab	9	Inpatient	Pneumonia	2013 Feb 15	73562 (Y)	3562	2c	Wild type
38814	Throat swab	11	Inpatient	Pneumonia	2013 Feb 15	73562 (Y)	3562	2c	Wild type
38941	Throat swab	33	Outpatient	Asymptomatic	2013 Feb 20	73563	3563	2c	No amp
38945	Throat swab	10	Inpatient	Pneumonia	2013 Feb 20	73563	3563	2c	No amp
38946	Throat swab	10	Inpatient	Pneumonia	2013 Feb 20	73563	3563	2c	Wild type
38960	Throat swab	33	Outpatient	Asymptomatic	2013 Feb 20	73563	3563	2c	Wild type
38962	Throat swab	51	Outpatient	Asymptomatic	2013 Feb 20	73562 (Y)	3562	2c	Wild type
39042	Throat swab	9	Inpatient	Pneumonia	2013 Feb 25	73562 (Y)	3562	2c	Wild type
39048	Throat swab	64	Outpatient	Asymptomatic	2013 Feb 25	63562 (V)	3562	2c	Wild type
39293	Throat swab	8	Inpatient	Pneumonia	2013 Mar 7	73562 (Y)	3562	2c	Wild type
Duchovshina									
43593	Throat swab	13	Inpatient	Pneumonia	2013 Oct 18	43562 (M)	3562	2c	Wild type
43596	Throat swab	15	Outpatient	Pneumonia	2013 Oct 18	43562 (M)	3562	2c	Wild type
43597	Throat swab	12	Inpatient	Pneumonia	2013 Oct 18	43562 (M)	3562	2c	Wild type
43692	Throat swab	5	Inpatient	Pneumonia	2013 Oct 22	43562 (M)	3562	2c	Wild type
43693	Throat swab	10	Inpatient	Pneumonia	2013 Oct 23	43562 (M)	3562	2c	Wild type
43694	Throat swab	9	Inpatient	Pneumonia	2013 Oct 22	43562 (M)	3562	2c	Wild type
43695	Throat swab	13	Inpatient	Pneumonia	2013 Oct 23	43562 (M)	3562	2c	Wild type
43804	Throat swab	9	Inpatient	Pneumonia	2013 Oct 27	43562 (M)	3562	2c	Wild type
43805	Throat swab	6	Inpatient	Pneumonia	2013 Oct 27	43562 (M)	3562	2c	Wild type
43806	Throat swab	14	Inpatient	Pneumonia	2013 Oct 27	43562 (M)	3562	2c	Wild type
43843	Throat swab	6	Inpatient	Pneumonia	2013 Oct 30	43562 (M)	3562	2c	Wild type
43888	Throat swab	58	Outpatient	Pneumonia	2013 Oct 31	43562 (M)	3562	2c	Wild type
43890	Throat swab	4	Inpatient	Pneumonia	2013 Oct 31	43562 (M)	3562	2c	Wild type
43919	Throat swab	41	Outpatient	Pneumonia	2013 Nov 1	43562 (M)	3562	2c	Wild type
43989	Throat swab	10	Inpatient	Pneumonia	2013 Nov 6	43562 (M)	3562	2c	Wild type
43990	Throat swab	13	Inpatient	Pneumonia	2013 Nov 6	43562 (M)	3562	2c	Wild type
43991	Throat swab	8	Inpatient	Pneumonia	2013 Nov 6	43562 (M)	3562	2c	Wild type
44174	Throat swab	10	Inpatient	Pneumonia	2013 Nov 14	43562 (M)	3562	2c	Wild type
44176	Throat swab	8	Inpatient	Pneumonia	2013 Nov 14	43562 (M)	3562	2c	No amp
Smolensk region									
89	Sputum	17	Inpatient	Pneumonia	2006 Oct 24	64572 (X)	4572	1	Wild type
95	Sputum	56	Inpatient	Pneumonia	2006 Oct 29	43562 (M)	3562	2a	Wild type
108–2	Sputum	19	Inpatient	Pneumonia	2006 Nov 3	34572 (J)	4572	1	Wild type
122	Sputum	18	Inpatient	Pneumonia	2006 Nov 18	64572 (X)	4572	1	Wild type
164	Sputum	39	Inpatient	Pneumonia	2007 Jan 25	34572 (J)	4572	1	Wild type
169	Sputum	43	Inpatient	Pneumonia	2007 Feb 4	43562 (M)	3562	2a	Wild type
175	Sputum	38	Inpatient	Pneumonia	2007 Feb 10	43562 (M)	3562	2a	Wild type
186	Sputum	48	Inpatient	Pneumonia	2007 Feb 20	4--62§	--62¶	2a	Wild type
187	Sputum	36	Inpatient	Pneumonia	2007 Feb 20	33562(G)	3562	2a	Wild type
192	Sputum	36	Inpatient	Pneumonia	2007 Feb 20	43562 (M)	3562	2a	Wild type
191	Sputum	40	Inpatient	Pneumonia	2007 Feb 24	43562 (M)	3562	2a	Wild type
211	Sputum	43	Inpatient	Pneumonia	2007 Mar 21	43562 (M)	3562	2a	Wild type
230	Sputum	17	Inpatient	Pneumonia	2007 Apr 17	43562 (M)	3562	2a	Wild type
257	Sputum	21	Inpatient	Pneumonia	2007 Jul 17	43672	3672	1	Wild type
261	Sputum	21	Inpatient	Pneumonia	2007 Jul 20	43672	3672	1	Wild type
270	Sputum	19	Inpatient	Pneumonia	2007 Jul 30	34572 (J)	4572	1	Wild type
271	Sputum	19	Inpatient	Pneumonia	2007 Jul 31	64572 (X)	4572	1	Wild type
280	Sputum	19	Inpatient	Pneumonia	2007 Aug 14	64572 (X)	4572	1	Wild type
293	Sputum	20	Inpatient	Pneumonia	2007 Sep 11	64572 (X)	4572	1	Wild type
298	Sputum	19	Inpatient	Pneumonia	2007 Sep 18	74572 (Z)	4572	1	Wild type
309	Sputum	36	Inpatient	Pneumonia	2007 Oct 02	23562 (B)	3562	2a	Wild type
310	Sputum	26	Inpatient	Pneumonia	2007 Oct 03	33562(G)	3562	2a	Wild type
313	Sputum	23	Inpatient	Pneumonia	2007 Oct 12	43562 (M)	3562	2a	Wild type
292	Sputum	17	Inpatient	Pneumonia	2007 Oct 22	33562(G)	3562	2a	Wild type
13	Throat swab	23	Inpatient	Pneumonia	2010 Feb 21	43562 (M)	3562	2c	Wild type
70	Throat swab	62	Inpatient	Pneumonia	2010 Mar 18	43562 (M)	3562	2a	Wild type
107	Throat swab	33	Inpatient	Pneumonia	2010 Mar 26	43562 (M)	3562	2a	Wild type
108–1	Throat swab	36	Inpatient	Pneumonia	2010 Mar 29	33562(G)	3562	2c	Wild type
212	Throat swab	67	Inpatient	Pneumonia	2010 Oct 15	24572 (E)	4572	1	Wild type

*MLVA, multilocus variable-number tandem-repeat; no amp, no amplification with the real-time PCR used to detect 23S rRNA mutations associated with macrolide resistance (*1*); RFLP, restriction fragment length polymorphism. An expanded version of this table is available online at http://wwwnc.cdc.gov/EID/article/22/2/15-1349-T1.htm. †The profiles are named according to a string of allele numbers in order of MPN1, MPN13, MPN14, MPN15, and MPN16 markers showing the number of repeats at each locus. When available, the naming according to Degrange et al. is shown in parentheses (*1*). ‡The profiles are named according to a string of allele numbers in order of MPN13, MPN14, MPN15 and MPN16 markers showing the number of repeats at each locus. The instable MPN1 marker (8) was removed. §Only 3 of 5 loci were amplified. -, indicates no amplification at second and third loci. ¶Only 2 of 4 loci were amplified. -, indicates no amplification at first and second loci.

For comparison purposes, because no previous data regarding *M. pneumoniae* molecular epidemiology in Russia were available, we retrospectively characterized 29 specimens, not from an outbreak, that were previously randomly collected for community-acquired pneumonia etiologic studies during October 2006–October 2007 and February–October 2010 by the laboratory of Smolensk State Medical Academy (Table). Of these specimens, 12 (41%) were P1 type 1, 15 (52%) were P1 type 2a, and only 2 (7%) were P1 type 2c. A polyclonal distribution with 8 distinct MLVA types was observed, with the MLVA type M representing 11 (38%) of the identified MLVA types. Without the MPN1 marker, 3 MLVA types were observed. No macrolide resistance–associated mutation was detected, similar to what was observed in the 32 specimens collected in 2013. This finding is consistent with the low prevalence of macrolide resistance reported in northern Europe (*6*,*7*).

We report 2 outbreaks of *M. pneumoniae* infections that occurred in the first and last quarter of 2013 in western Russia (Smolensk region). Despite the high predominance of P1 type 1 strains reported in the recent literature (*1*,*2*,*7*), these 2 outbreaks, reported in semiclosed settings involved only the newly described P1 type 2c variant; 1 outbreak represented a monoclonal phenomenon. In the Smolensk region, the circulation of both type 1 and 2 strains was observed a few years before the outbreak; most of these strains were P1 type 2a variants, and only a minority were type 2c variants, suggesting that the new type 2c variant had spread throughout this region of Russia since at least 2006. In other parts of the world, a switch between type 1 and type 2 strains might be occurring. Indeed, in the United States, P1 type 1 isolates predominated before 2010 but dropped to 50% of isolates in 2013, and type 2 and type 2 variant strains increased (*9*). This cyclic pattern of type 1 or type 2 predominance in the population has previously been reported (*10*).

In conclusion, we detected no macrolide resistance in western Russia. The P1 type 2c variant spread throughout this region and can be responsible for monoclonal outbreaks. The epidemiologic monitoring of *M. pneumoniae* P1 types will assess the potential switch to P1 type 2 in the United States and other parts of the world and detect the possible emergence of the P1 type 2c variant.
